# Genetic and environmental determinants of human TCR repertoire diversity

**DOI:** 10.1186/s12979-020-00195-9

**Published:** 2020-09-04

**Authors:** Chirag Krishna, Diego Chowell, Mithat Gönen, Yuval Elhanati, Timothy A. Chan

**Affiliations:** 1 Computational and Systems Biology Program, Memorial Sloan Kettering Cancer Center, New York, NY 10065 USA; 2 Human Oncology and Pathogenesis Program, Memorial Sloan Kettering Cancer Center, New York, NY 10065 USA; 3 Department of Epidemiology and Biostatistics, Sloan Kettering Institute for Cancer Research, New York, NY 10065 USA; 4 Immunogenomics and Precision Oncology Platform, Memorial Sloan Kettering Cancer Center, New York, NY 10065 USA; 5 Department of Radiation Oncology, Memorial Sloan Kettering Cancer Center, New York, NY 10065 USA; 6 Weill Cornell School of Medicine, New York, NY 10065 USA; 7 Center for Immunotherapy and Precision Immuno-Oncology, Cleveland Clinic, Cleveland, OH 44195 USA

**Keywords:** Major histocompatibility complex, Heterozygote advantage, T cell receptor repertoire, Infection, Aging, Immunogenetics

## Abstract

T cell discrimination of self and non-self is the foundation of the adaptive immune response, and is orchestrated by the interaction between T cell receptors (TCRs) and their cognate ligands presented by major histocompatibility (MHC) molecules. However, the impact of host immunogenetic variation on the diversity of the TCR repertoire remains unclear. Here, we analyzed a cohort of 666 individuals with TCR repertoire sequencing. We show that TCR repertoire diversity is positively associated with polymorphism at the human leukocyte antigen class I (HLA-I) loci, and diminishes with age and cytomegalovirus (CMV) infection. Moreover, our analysis revealed that HLA-I polymorphism and age independently shape the repertoire in healthy individuals. Our data elucidate key determinants of human TCR repertoire diversity, and suggest a mechanism underlying the evolutionary fitness advantage of HLA-I heterozygosity.

## Background

The large sequence diversity of the TCR repertoire is a hallmark of the adaptive immune system, and varies markedly across individuals [1–4]. This diversity, estimated to exceed 10^6^ sequences in humans [5–7], is shaped by stochastic [8] and genetic [9] effects in conjunction with continuous immunological challenges throughout life [9]. In the thymus, VDJ recombination facilitates random rearrangement of the complementary determining region 3 (CDR3) within the TCR α and β loci, followed by random nucleotide insertion and deletions at junction sites [10]. The CDR3 regions of the TCR are primarily responsible for interacting with the peptide presented by MHC [11], with the potential diversity of CDR3β exceeding that of CDR3α [12]. Whether a particular TCR joins the periphery depends on its behavior during thymic selection, in which TCRs interact with both self peptide and MHC [13, 14]. TCRs that fail to bind to peptide-MHC complexes and those that bind too strongly are eliminated [15, 16]. Those TCRs that survive thymic selection are responsible for mounting productive immune responses through continuous interaction with self and foreign peptides bound to MHC molecules. TCR diversity can determine how efficiently one rejects pathogens such as viruses, and potentially cancer cells. Accordingly, considerable effort has been devoted to understanding how MHC genetic variation impacts the TCR repertoire.

MHC restriction is the cornerstone of T cell recognition [17], and prior reports have assessed the effect of the presence of specific MHC alleles on TCR V gene usage [18, 19] and repertoire sharing [9, 20]. These data, together with structural studies of the TCR-MHC interface [11, 21–24], have provided key insights into how the TCR binds MHC and peptide. However, it remains unknown to what extent HLA polymorphism affects TCR repertoire diversity in humans.

## Results

**Fig. 1:**
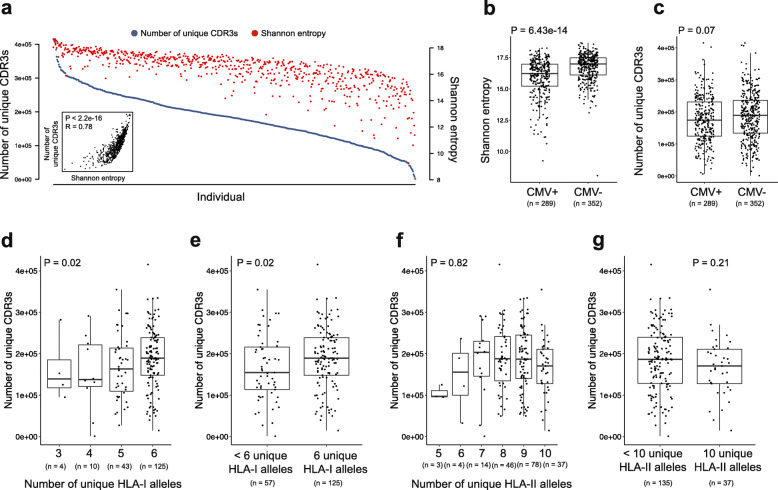
CMV serostatus and HLA-I genotype are associated with TCR repertoire diversity. **a** Variation in number of unique CDR3s and Shannon entropy, two measures of TCR repertoire diversity, across the cohort. **b** Association of CMV seropositivity (CMV+) with reduced TCR repertoire diversity (Shannon entropy). *P* = 6.43e-14, two-sided Wilcoxon test. **c** Association of CMV seropositivity (CMV+) with reduced TCR repertoire diversity (number of unique CDR3s). *P* = 0.07, two-sided Wilcoxon test. **d** Association of HLA-I polymorphism with increased number of unique CDR3s in CMV- individuals; HLA-I *P* = 0.02, estimate = 18,787.8; age *P* = 0.002, estimate − 1326.3. *P*-values are from a linear model incorporating number of unique HLA-I alleles and age. **e** Association of full HLA-I heterozygosity (6 different HLA-I alleles) with number of unique CDR3s in CMV- individuals; full HLA-I heterozygosity *P =* 0.02, estimate = 29,248.4; age *P =* 0.002, estimate = − 1342.6. *P-*values are from a linear model incorporating a binary variable encoding full HLA-I heterozygosity, and age as a continuous variable. **f** No association between HLA-II polymorphism and number of unique CDR3s in CMV- individuals; HLA-II *P* = 0.82, estimate = 1224.9; age *P* = 0.006, estimate = − 1182.1. *P-*values are from in a linear model incorporating number of unique HLA-II alleles and age. **g** No association between full HLA-II heterozygosity (10 unique HLA-II alleles) and number of unique CDR3s in CMV- individuals; HLA-II *P* = 0.21, estimate = − 17,362.9; age *P =* 0.006, estimate = − 1153.4. *P-*values are from a linear model incorporating a binary variable encoding full HLA-II heterozygosity, and age as a continuous variable

We sought to address this question. Thus, we studied a cohort of 666 individuals, with annotated CMV serostatus, ethnicity, age, sex, high-resolution HLA class I and class II genotypes, and bulk TCRβ sequencing from PBMCs [9, 25] (Additional file 1: Table S1). 85% of the individuals were white, 52% were male, and 45% were female, with the remainder of unknown sex. We first quantified TCR repertoire diversity by applying two measures widely used in repertoire and ecological studies—the number of unique CDR3β amino acid sequences (a.k.a. richness), and Shannon entropy, a diversity measure that is weighted by the abundance of each CDR3 [26]. We found both measures to be highly correlated, and observed high variability in TCR repertoire diversity across the cohort (Fig. 1a; richness range 1055–415,509, Shannon entropy range 8.1–18.7; R = 0.78, *P* < 0.0001). Accordingly, we anticipated that CMV—a chronic infection prevalent in 30–90% of adults [27] and a model system for the study of public T cell responses [28]—would be a key determinant of the observed wide variation in repertoire diversity. Indeed, individuals with CMV (CMV+) exhibited a reduction in TCR repertoire diversity compared to those without (CMV-) (Fig. 1b-c). This reduction was most striking when using Shannon entropy (*P* < 0.0001, Wilcoxon test; Fig. 1b), consistent with prior work demonstrating that CMV alters the diversity, but not overall size of the CD8+ T cell response [29]. Altogether, these data suggest that CMV diminishes TCR repertoire diversity, and are in line with a recent study demonstrating dramatic reduction of the antibody repertoire after measles infection [30]—highlighting the need for widespread and continuous vaccination against infectious disease.

**Fig. 2:**
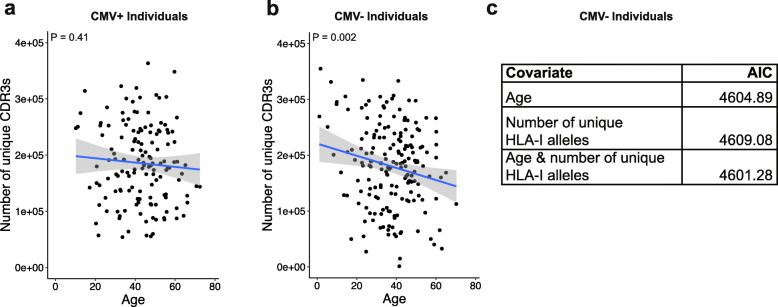
Age and HLA-I polymorphism independently affect TCR repertoire diversity in CMV- individuals. **a** No association between age and number of unique CDR3s in CMV+ individuals; age *P* = 0.41, estimate = − 378.5; HLA-I *P* = 0.70, estimate = − 3318.9. *P-*values are from a linear model incorporating age and number of unique HLA-I alleles. **b** Association between age and number of unique CDR3s in CMV- individuals; age *P =* 0.002, estimate = − 1326.3; HLA-I *P =* 0.02, estimate = 18,787.8. *P-*values are from a linear model incorporating age and number of unique HLA-I alleles. **c** AIC analysis of three linear models with number of unique CDR3s as the dependent variable, and either age alone, number of unique HLA-I alleles alone, or both as the independent variables. All models were fit in CMV- individuals. Data show that the best model that explains the observed TCR repertoire diversity across these individuals is the one with both age and number of unique HLA-I alleles (AIC = 4601.28)

We next limited our analyses to individuals with complete HLA-I (HLA-A, B, & C) and II (HLA-DRB, DPB, DQB, DQA, & DPA) genotypes, and given the impact of CMV on the TCR repertoire described above, considered CMV+ and CMV- individuals separately (Additional file 2: Fig. S1). We used a linear model to test the association between HLA polymorphism—measured here as the number of different HLA-I alleles in each individual—and TCR repertoire diversity in CMV- individuals. Strikingly, we observed that TCR repertoire diversity was positively associated with the number of HLA-I alleles (*P* = 0.02; Fig. 1d). Furthermore, we observed that CMV- individuals fully heterozygous at HLA-I genes had higher TCR repertoire diversity than individuals who were homozygous at least in one HLA-I locus (*P =* 0.02; Fig. 1e). We found the same associations when considering Shannon entropy instead of richness (Additional file 2: Fig. S2a-b). Importantly, these results were independent of age, previously shown to be negatively correlated with TCR repertoire diversity [31–33] and shown here to be independent of the number of HLA-I alleles. Interestingly, we found no association between HLA-II polymorphism and TCR repertoire diversity (Fig. 1f-g and Additional file 2: Fig. S2c-d). These data may suggest that heterozygosity at HLA-II may be disadvantageous given the strong associations between many HLA-II haplotypes and susceptibility to autoimmune disease [34]. Finally, we repeated these analyses in CMV+ individuals, and observed no association between HLA polymorphism and TCR repertoire diversity (Additional file 2: Fig. S3). Notably, we found no association between age and number of unique CDR3s in CMV+ individuals either (*P* = 0.41; Fig. 2a), whereas in CMV- negative individuals, we observed that the number of unique CDR3s diminished with age (*P* = 0.002; Fig. 2b). When considering Shannon entropy instead of richness, the effect of age was weaker in CMV+ individuals (*P* = 0.03; Additional file 2: Fig. S4a) than in CMV- individuals (*P* = 0.0005; Additional file 2: Fig. S4b). These results suggest a dominant role of chronic infection over host genetics and age in significantly altering the TCR repertoire. However, HLA diversity may affect antigen-specific TCRs rather than the whole repertoire as suggested by past studies [35], and should be the subject of future complementary analyses focused on the diversity of CMV-specific expanded clones.

We next sought to assess the combined effect of age and HLA-I polymorphism on repertoire diversity. We developed three separate linear models in CMV- individuals—one with age alone, one with number of unique HLA-I alleles alone, and one with both age and number of unique HLA-I alleles. We selected the most strongly supported model based on the Akaike information criterion (AIC), i.e., the best-fit model yields the smallest AIC value [36]. We found that the best model that explained the observed TCR repertoire diversity in these individuals included both variables, (AIC = 4601.28; Fig. 2c; AIC = 575.11, Additional file 2: Fig. S4c). As expected, the combined effect of age and HLA-I polymorphism was not observed in CMV+ individuals (Additional file 2: Fig. S4d).

Recent studies have demonstrated that HLA-I evolutionary divergence (HED), a continuous and granular metric of HLA-I polymorphism, measures the breadth of the immunopeptidome bound by an individual’s MHC-I molecules [37, 38]. Therefore, we sought to investigate the association between mean HED, an aggregate measure of HED across the three classical HLA-I loci [37, 38], and TCR repertoire diversity. High mean HED—defined here as mean HED greater than the median—was associated with increased TCR repertoire diversity in CMV- individuals, and was independent of age (*P* = 0.03; Additional file 2: Fig. S5a). These results suggest that individuals with more divergent HLA genotypes, and correspondingly broader immunopeptidomes, have increased TCR repertoire diversity. Moreover, consistent with our earlier analysis, the best model of TCR repertoire diversity included both high mean HED and age (AIC = 577.6; Additional file 2: Fig. S5b). This effect was not observed in CMV+ individuals (Additional file 2: Fig. S5c-d). These data thus provide further evidence for the notion that TCR repertoire diversity is increased in individuals with greater HLA diversity.

## Conclusions

To our knowledge, our study is the first to show empirically that HLA-I polymorphism increases TCR repertoire diversity in humans, and has several important implications. First, our results add an important dimension to the HLA heterozygote advantage hypothesis, which states that HLA-heterozygous individuals present a broader immunopeptidome for recognition by cytotoxic T cells [17, 35, 37–41]. In particular, our data suggest that an additional potential consequence of enhanced peptide presentation in HLA-heterozygous individuals is a more diverse TCR repertoire, which could improve immune protection and evolutionary fitness.

An important consideration is the direction of the association between HLA polymorphism and TCR repertoire diversity, which has remained controversial. The TCR depletion hypothesis suggests that increasing individual MHC polymorphism—leading to more self peptides presented during negative selection—creates “holes” in the TCR repertoire, thereby decreasing its diversity in the periphery [42–45]. However, there is no experimental evidence for the TCR depletion hypothesis in humans. Our results may suggest a dominant role for positive selection in influencing TCR repertoire diversity. This idea is in line with prior theoretical work suggesting that additional MHC variants enhance positive selection, and consequently the number of T cells that can survive negative selection [46]. Indeed, our study motivates empirical investigation of how each step of thymic selection affects the TCR repertoire in an MHC-dependent fashion, which remains unclear.

Finally, while our study demonstrates that HLA-I polymorphism, age, and chronic infection shape the TCR repertoire, a full account of the determinants of TCR repertoire diversity remains unknown. Of note, variation in TCR repertoire diversity across individuals may be driven in part by differences in T cell sampling. The sample size variation in our cohort spans an order of magnitude, and may be driven in part by uncontrolled factors in the sequencing process. As a possible control for sample size variation, we quantified TCR repertoire diversity in CMV- individuals using a normalized form of the Shannon entropy, (Methods). Using this slightly corrected measure, we still observed a positive association between HLA-I polymorphism and TCR repertoire diversity (Additional file 2: Fig. S6). Indeed, our analyses suggest that despite confounding variation in TCR repertoire sample sizes across individuals, biological factors such as age and HLA-I polymorphism independently affect TCR repertoire diversity. In addition, prior studies have suggested that the TCR repertoire differs by sex [42, 47]. We also detected a trend towards reduced TCR repertoire diversity in CMV- males in our cohort (*P* = 0.09, Additional file 2: Fig. S7). However, larger numbers may be required to clarify this association. Future work may investigate how the TCR repertoire is shaped by vaccination, or how HLA polymorphism and TCR repertoire diversity act in concert to influence overall mortality.

## Methods

### Cohort assembly

We analyzed all individuals in the cohort from Emerson et al and Dewitt III et al [9, 25] (Additional file 1: Table S1)*.* This cohort represents the largest dataset generated to date with bulk TCRβ-sequencing from PBMCs and 4-digit HLA-I & II genotypes. Full details of the TCRβ-sequencing and HLA genotyping are available in the original studies. Briefly, CMV serostatus was tested at Fred Hutchinson Cancer Center following protocol approval by an institutional review board, and written informed consent. The CDR3 region of the TCRβ locus was amplified and sequenced from PBMCs as described previously [6] (raw files available at https://clients.adaptivebiotech.com/pub/Emerson-2017-NatGen). The HLA genotypes for these individuals were generated and validated via molecular typing methods (specific oligonucleotide probe typing, Sanger sequencing, or next generation sequencing) together with SNP imputation in DeWitt/Bradley et al [9] (raw files available at doi:10.5281/zenodo.1248193). In particular, their study genotyped the 3 classical HLA-I loci (HLA-A, B, & C), and 5 HLA-II loci (HLA-DRB, DPB, DQB, DQA, & DPA). Thus, the range of unique HLA-I alleles for HLA-I was 3 (fully homozygous) to 6 (fully heterozygous), and 5–10 for HLA-II.

### Calculation of repertoire diversity metrics

Two metrics were used to measure TCR repertoire diversity- richness and Shannon entropy. For richness, we counted the total number of unique productive CDR3β amino acid sequences for each individual. The Shannon entropy of each individual’s repertoire was calculated using all CDR3β sequences, defined as:

$$ H=-{\sum}_sf(s)\ \log\ f(s) $$

where the sum is taken over all clones *s* and *f*(*s*) is the frequency of clone *s*. The normalized Shannon-Wiener index was calculated using the vdjtools package [48].

### Calculation of HLA evolutionary divergence

HLA evolutionary divergence (HED) was calculated for each individual as described previously [37, 38]. First, the protein sequences corresponding to the peptide binding domain of each allele of each patient’s HLA-I genotype (exons 2 and 3, obtained from the ImMunoGeneTics/HLA [49] and Ensembl [50] databases) were extracted. The divergence between allele sequences was calculated using the Grantham distance [51], which considers the physiochemical properties of amino acids, and thus the functional similarity between sequences. First, given a particular HLA-I locus, the sequences of the peptide-binding domains of each allele are aligned [52] and the Grantham distance is calculated as the sum of amino acid differences along the sequences of the peptide-binding domains:

$$ \mathrm{Grantham}\ \mathrm{Distance}=\sum {\mathrm{D}}_{ij}=\sum {\left[\alpha {\left({c}_i-{c}_j\right)}^2+\beta {\left({p}_i-{p}_j\right)}^2+\gamma {\left({v}_i-{v}_j\right)}^2\right]}^{1/2} $$

where *i* and *j* are the two homologous amino acids at a given position in the alignment and D is the Grantham distance between them. *c, p* and *v* represent composition, polarity and volume of the amino acids, respectively, and *α*, *β* and *γ* are constants. All values are taken from the original study. The final Grantham distance is calculated by normalizing the value from equation above by the length of the alignment between the peptide-binding domains of a particular HLA-I genotype’s two alleles. An analysis presented in Pierini & Lenz of multiple common sequence divergence measures showed that the correlation of Grantham distance with the number of peptides bound by both alleles of a heterozygous genotype exceeded that of the other distance measures. Mean HED was calculated as the mean of divergences at HLA-A, HLA-B and HLA-C.

### Statistical analyses

All analyses involving associations between number of unique HLA-I & II alleles and TCR repertoire diversity were conducted using a linear model with the lm() function in the R Statistical Computing Environment v3.6.1 (http://www.r-project.org). The numbers of unique HLA-I and II alleles were considered ordinal data for linear modeling. Akaike Information Criteria (AIC) for comparisons of linear models with age and/or HLA-I polymorphism were calculated using the AIC() function in R.

## Supplementary information

**Additional file 1: Table S1.** Individuals from Emerson et al and Dewitt III et al and all variables analyzed in the present study.

**Additional file 2: Fig. S1.** Cohort assembly and filtering. Flowchart depicting the studies in which TCR sequencing and HLA genotyping were performed, and steps used to select individuals for analysis. **Fig. S2.** Association of HLA-I and II polymorphism with TCR repertoire Shannon entropy in CMV- individuals. **a** Association of HLA-I polymorphism with increased Shannon entropy in CMV- individuals; HLA-I *P* = 0.008, estimate = 0.33; age *P* = 0.0005, estimate = -0.02. *P*-values are from a linear model incorporating the number of unique HLA-I alleles and age. **b** Association of full HLA-I heterozygosity (6 unique HLA-I alleles) with increased Shannon entropy; full HLA-I heterozygosity *P* = 0.01, estimate = 0.46; age *P* = 0.0005, estimate = -0.02. *P*-values are from a linear model incorporating a binary variable encoding full HLA-I heterozygosity, and age as a continuous variable. **c** No association between HLA-II polymorphism and Shannon entropy; HLA-II *P* = 0.24, estimate = 0.1; age *P* = 0.002, estimate = -0.02. *P*-values are from a linear model incorporating number of unique HLA-II alleles and age. **d** No association between full HLA-II heterozygosity (10 unique HLA-II alleles) and Shannon entropy; full HLA-II heterozygosity *P* = 0.65, estimate = -0.10; age *P* = 0.002, estimate = -0.02. *P*-values are from a linear model incorporating a binary variable encoding full HLA-II heterozygosity, and age as a continuous variable. **Fig. S3.** Neither HLA-I nor HLA-II polymorphism is associated with TCR repertoire diversity in CMV+ individuals. **a** No association between HLA-I polymorphism and number of unique CDR3s; HLA-I *P* = 0.70., estimate = -3318.9; age *P* = 0.41, estimate = -378.5. *P*-values are from a linear model incorporating the number of unique HLA-I alleles and age. **b** No association between HLA-I polymorphism and Shannon entropy; HLA-I *P* = 0.80, estimate = -0.04; age *P* = 0.03, estimate = -0.02. *P*-values are from a linear model incorporating the number of unique HLA-I alleles and age. **c** No association between HLA-II polymorphism and number of unique CDR3s; HLA-II *P* = 0.45, estimate = 3918.7; age *P* = 0.37, estimate = -414.3. *P*-values are from a linear model incorporating the number of unique HLA-II alleles and age. **d** No association between HLA-II polymorphism and Shannon entropy; HLA-II *P* = 0.70, estimate = 0.04; age *P* = 0.03, estimate = -0.02. *P*-values are from a linear model incorporating the number of unique HLA-II alleles and age. **Fig. S4.** Age and HLA-I polymorphism independently affect TCR repertoire Shannon entropy in CMV- individuals. **a** Association between age and Shannon entropy in CMV+ individuals; age *P* = 0.03, estimate = -0.02; HLA-I *P* = 0.80, estimate = -0.04. *P*-values are from a linear model incorporating age and number of unique HLA-I alleles. **b** Association between age and Shannon entropy in CMV- individuals; age *P* = 0.0005, estimate = -0.02; HLA-I *P* = 0.008, estimate = 0.33. *P*-values are from a linear model incorporating age and number of unique HLAI alleles. **c** AIC analysis of three linear models with Shannon entropy as the dependent variable, and either age alone, number of unique HLA-I alleles alone, or both as the independent variables. All models were fit in CMV- individuals. Data show that the best model that explains the observed TCR repertoire diversity across these individuals is the one with both age and number of unique HLA-I alleles (AIC = 575.11). **d** AIC analysis of three linear models with Shannon entropy as the dependent variable, and either age alone, number of unique HLA-I alleles alone, or both as the independent variables. All models were fit in CMV+ individuals. Data show that number of unique HLA-I alleles adds no effect beyond the effect of age alone. **Fig. S5.** Mean HLA evolutionary divergence is associated with increased TCR repertoire diversity in CMV- individuals. **a** Association of high mean HED (Mean HED >= median) with increased Shannon entropy in CMV- individuals; high mean HED *P* = 0.03, estimate = 0.37; age *P* = 0.001, estimate = -0.02. *P*-values are from a linear model incorporating corresponds a binary variable encoding high mean HED, and age as a continuous variable. **b** AIC analysis of three linear models with number of unique CDR3s as the dependent variable, and either age alone, high mean HED alone, or both as the independent variables. All models were fit in CMV- individuals. Data show that the best model that explains the observed TCR repertoire diversity across these individuals is the one with both age and high mean HED (AIC = 577.6). **c** No association of high mean HED (Mean HED >= median) with Shannon entropy in CMV+individuals; high mean HED *P* = 0.87, estimate = -0.04; age *P* = 0.03, estimate = -0.02. *P*-values are from a linear model incorporating a binary variable encoding high mean HED, and age as a continuous variable. **d** AIC analysis of three linear models with Shannon entropy as the dependent variable, and either age alone, high mean HED alone, or both as the independent variables. All models were fit in CMV+ individuals. Data show that high mean HED adds no effect beyond the effect of age alone. **Fig. S6.** Association of HLA-I diversity with TCR repertoire diversity measured using the normalized Shannon-Wiener index in CMV- individuals. **a** Association of HLA-I polymorphism with normalized Shannon-Wiener index in CMV- individuals; HLA-I *P* = 0.07, estimate = 0.006; age *P* = 0.0002, estimate -0.0007. *P*-values are from a linear model incorporating number of unique HLA-I alleles and age. **b** AIC analysis of three linear models with TCR normalized Shannon-Wiener index as the dependent variable, and either age alone, number of unique HLA-I alleles alone, or both as the independent variables. Data show that the best model that explains the observed TCR repertoire diversity across these individuals is the one with both age and number of unique HLA-I alleles (AIC = -727.0241). **c** Association of high mean HED (Mean HED >= median) with increased normalized Shannon-Wiener index in CMV- individuals; high mean HED *P* = 0.01, estimate = 0.01; age *P* = 0.01, estimate = -0.0006. *P*-values are from a linear model incorporating corresponds a binary variable encoding high mean HED, and age as a continuous variable. **d** AIC analysis of three linear models with TCR normalized Shannon-Wiener index as the dependent variable, and either age alone, high mean HED alone, or both as the independent variables. Data show that the best model that explains the observed TCR repertoire diversity across these individuals is the one with both age and high mean HED (AIC = -729.9617). **Fig. S7.** Association of sex with TCR repertoire diversity in CMV- individuals. **a** Linear model testing the association of sex with number of unique CDR3s in CMV- individuals. **b** Linear model testing the association of sex with Shannon entropy in CMV- individuals.

## Data Availability

All supporting data are including as supplementary material.
