# Supplementary material for: Genetic and environmental determinants of human TCR repertoire diversity
Source: Immun Ageing. 2020 Sep 4;17:26. doi: 10.1186/s12979-020-00195-9 (PMC7487954; doi:10.1186/s12979-020-00195-9)
Supplement: Supplementary file 2 — Additional file 2: Fig. S1. Cohort assembly and filtering. Flowchart depicting the studies in which TCR sequencing and HLA genotyping were performed, and steps used to select individuals for analysis. Fig. S2. Association of HLA-I and II polymorphism with TCR repertoire Shannon entropy in CMV- individuals. a Association of HLA-I polymorphism with increased Shannon entropy in CMV- individuals; HLA-I P = 0.008, estimate = 0.33; age P = 0.0005, estimate = -0.02. P-values are from a linear model incorporating the number of unique HLA-I alleles and age. b Association of full HLA-I heterozygosity (6 unique HLA-I alleles) with increased Shannon entropy; full HLA-I heterozygosity P = 0.01, estimate = 0.46; age P = 0.0005, estimate = -0.02. P-values are from a linear model incorporating a binary variable encoding full HLA-I heterozygosity, and age as a continuous variable. c No association between HLA-II polymorphism and Shannon entropy; HLA-II P = 0.24, estimate = 0.1; age P = 0.002, estimate = -0.02. P-values are from a linear model incorporating number of unique HLA-II alleles and age. d No association between full HLA-II heterozygosity (10 unique HLA-II alleles) and Shannon entropy; full HLA-II heterozygosity P = 0.65, estimate = -0.10; age P = 0.002, estimate = -0.02. P-values are from a linear model incorporating a binary variable encoding full HLA-II heterozygosity, and age as a continuous variable. Fig. S3. Neither HLA-I nor HLA-II polymorphism is associated with TCR repertoire diversity in CMV+ individuals. a No association between HLA-I polymorphism and number of unique CDR3s; HLA-I P = 0.70., estimate = -3318.9; age P = 0.41, estimate = -378.5. P-values are from a linear model incorporating the number of unique HLA-I alleles and age. b No association between HLA-I polymorphism and Shannon entropy; HLA-I P = 0.80, estimate = -0.04; age P = 0.03, estimate = -0.02. P-values are from a linear model incorporating the number of unique HLA-I alleles and age. c No associa [file 12979_2020_195_MOESM2_ESM.zip › FigS5.pdf]

Figure S5

a

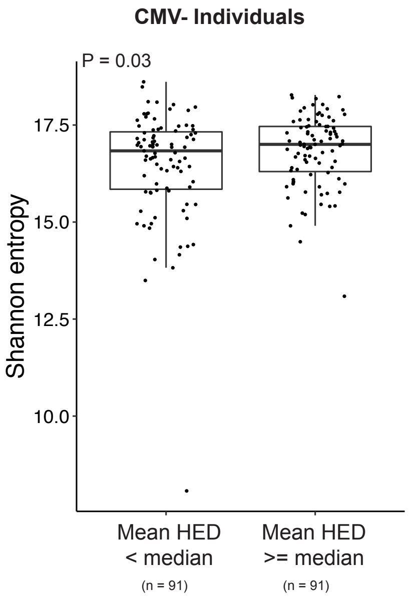

b

CMV- Individuals

Covariate	AIC
Age	580.3
Mean HED >= median	586
Age & mean HED >= median	577.6

c

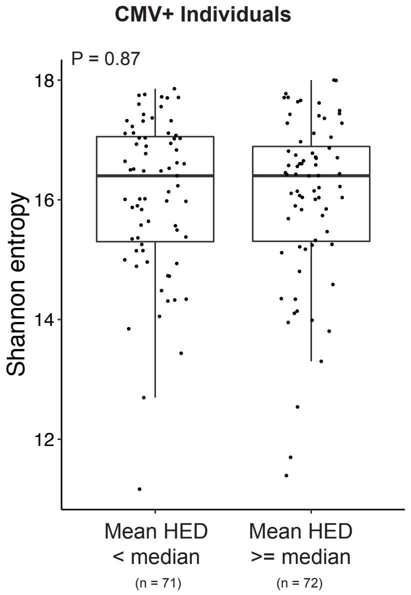

d

CMV+ Individuals

Covariate	AIC
Age	493.66
Mean HED >= median	498.65
Age & mean HED >= median	495.6
