# Supplementary material for: Genetic and environmental determinants of human TCR repertoire diversity
Source: Immun Ageing. 2020 Sep 4;17:26. doi: 10.1186/s12979-020-00195-9 (PMC7487954; doi:10.1186/s12979-020-00195-9)
Supplement: Supplementary file 2 — Additional file 2: Fig. S1. Cohort assembly and filtering. Flowchart depicting the studies in which TCR sequencing and HLA genotyping were performed, and steps used to select individuals for analysis. Fig. S2. Association of HLA-I and II polymorphism with TCR repertoire Shannon entropy in CMV- individuals. a Association of HLA-I polymorphism with increased Shannon entropy in CMV- individuals; HLA-I P = 0.008, estimate = 0.33; age P = 0.0005, estimate = -0.02. P-values are from a linear model incorporating the number of unique HLA-I alleles and age. b Association of full HLA-I heterozygosity (6 unique HLA-I alleles) with increased Shannon entropy; full HLA-I heterozygosity P = 0.01, estimate = 0.46; age P = 0.0005, estimate = -0.02. P-values are from a linear model incorporating a binary variable encoding full HLA-I heterozygosity, and age as a continuous variable. c No association between HLA-II polymorphism and Shannon entropy; HLA-II P = 0.24, estimate = 0.1; age P = 0.002, estimate = -0.02. P-values are from a linear model incorporating number of unique HLA-II alleles and age. d No association between full HLA-II heterozygosity (10 unique HLA-II alleles) and Shannon entropy; full HLA-II heterozygosity P = 0.65, estimate = -0.10; age P = 0.002, estimate = -0.02. P-values are from a linear model incorporating a binary variable encoding full HLA-II heterozygosity, and age as a continuous variable. Fig. S3. Neither HLA-I nor HLA-II polymorphism is associated with TCR repertoire diversity in CMV+ individuals. a No association between HLA-I polymorphism and number of unique CDR3s; HLA-I P = 0.70., estimate = -3318.9; age P = 0.41, estimate = -378.5. P-values are from a linear model incorporating the number of unique HLA-I alleles and age. b No association between HLA-I polymorphism and Shannon entropy; HLA-I P = 0.80, estimate = -0.04; age P = 0.03, estimate = -0.02. P-values are from a linear model incorporating the number of unique HLA-I alleles and age. c No associa [file 12979_2020_195_MOESM2_ESM.zip › FigS7.pdf]

Figure S7

a

Number of unique CDR3s

Covariate	Estimate	Standard Error	t value	P-value
(Intercept)	149274.6	47114.3	3.168	0.0018
Sex (M)	-18945.6	11234.6	-1.686	0.09
Age	-1310.3	419.4	-3.124	0.002
Number of unique HLA-I alleles	16488.3	8012.3	2.058	0.04

b

Shannon entropy

Covariate	Estimate	Standard Error	t value	P-value
(Intercept)	16.03071	47114.3	3.168	0.0018
Sex (M)	-0.29858	11234.6	-1.686	0.09
Age	-0.02329	419.4	-3.124	0.002
Number of unique HLA-I alleles	0.29872	0.1259	2.373	0.01873
